# The ER-Mitochondria Tethering Complex VAPB-PTPIP51 Regulates Autophagy

**DOI:** 10.1016/j.cub.2016.12.038

**Published:** 2017-02-06

**Authors:** Patricia Gomez-Suaga, Sebastien Paillusson, Radu Stoica, Wendy Noble, Diane P. Hanger, Christopher C.J. Miller

**Affiliations:** 1 Department of Basic and Clinical Neuroscience, Institute of Psychiatry, Psychology and Neuroscience, King’s College London, London SE5 9RX, UK

**Keywords:** autophagy, endoplasmic reticulum, mitochondria, PTPIP51, VAPB, Alzheimer’s disease, Parkinson’s disease, amyotrophic lateral sclerosis, calcium, MAM

## Abstract

Mitochondria form close physical associations with the endoplasmic reticulum (ER) that regulate a number of physiological functions. One mechanism by which regions of ER are recruited to mitochondria involves binding of the ER protein VAPB to the mitochondrial protein PTPIP51, which act as scaffolds to tether the two organelles. Here, we show that the VAPB-PTPIP51 tethers regulate autophagy. We demonstrate that overexpression of VAPB or PTPIP51 to tighten ER-mitochondria contacts impairs, whereas small interfering RNA (siRNA)-mediated loss of VAPB or PTPIP51 to loosen contacts stimulates, autophagosome formation. Moreover, we show that expression of a synthetic linker protein that artificially tethers ER and mitochondria also reduces autophagosome formation, and that this artificial tether rescues the effects of siRNA loss of VAPB or PTPIP51 on autophagy. Thus, these effects of VAPB and PTPIP51 manipulation on autophagy are a consequence of their ER-mitochondria tethering function. Interestingly, we discovered that tightening of ER-mitochondria contacts by overexpression of VAPB or PTPIP51 impairs rapamycin- and torin 1-induced, but not starvation-induced, autophagy. This suggests that the regulation of autophagy by ER-mitochondria signaling is at least partly dependent upon the nature of the autophagic stimulus. Finally, we demonstrate that the mechanism by which the VAPB-PTPIP51 tethers regulate autophagy involves their role in mediating delivery of Ca^2+^ to mitochondria from ER stores. Thus, our findings reveal a new molecular mechanism for regulating autophagy.

## Highlights

• Loosening ER-mitochondria contacts by loss of VAPB-PTPIP51 stimulates autophagy
• Tightening ER-mitochondria contacts by increased VAPB-PTPIP51 inhibits autophagy
• Artificial ER-mitochondria tethers rescue VAPB-PTPIP51 loss effects on autophagy
• The effects of VAPB-PTPIP51 involve their role in ER-mitochondria Ca^2+^ delivery

## Graphical Abstract

**Figure:**
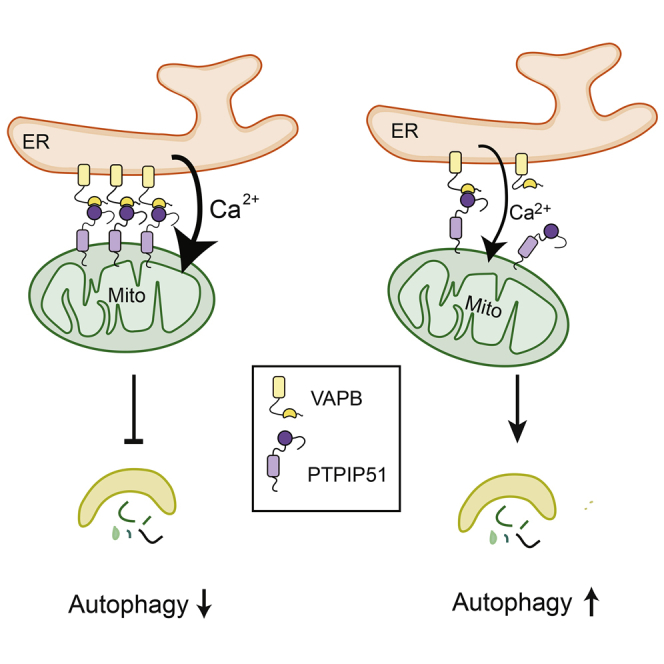

## Teaser

Tight contacts between ER and mitochondria facilitate IP3-receptor-mediated delivery of Ca^2+^ to mitochondria. VAPB and PTPIP51 are tethering proteins that mediate formation of these contacts. Gomez-Suaga et al. show that the VAPB-PTPIP51 tethers regulate autophagy and that this involves their role in facilitating ER-mitochondria Ca^2+^ exchange.

## Introduction

Macroautophagy, hereafter termed autophagy, is an evolutionarily conserved cellular process by which cytosolic constituents, including damaged organelles and aggregated proteins, are engulfed within specialized double-membrane vesicles known as autophagosomes. These then fuse with the endosomal-lysosomal system, and this facilitates degradation of their contents to yield metabolites that can be released into the cytoplasm for recycling [1]. Autophagy occurs at basal levels in virtually all cells, and this autophagic flux permits the removal of cellular components that accumulate during normal cell functions [1]. In addition, autophagy provides a mechanism by which cell components are removed in certain physiological states, such as during development and following nutrient starvation, but is also a key process in some diseases. Indeed, alterations to autophagy are believed to contribute to cancer and neurodegenerative diseases [2].

Autophagosome formation commences with the development of an initial cup-shaped isolation membrane known as the phagophore, which expands to progressively engulf the cytosolic material destined for degradation [1]. Once the phagophore membrane has sealed to surround the target material, the autophagosome fuses with a lysosome (or endosome and then a lysosome) to form an autolysosome, and the contents are then digested to yield metabolites that can be released into the cytoplasm for recycling [1]. Although many aspects of the autophagic process are now becoming clear, the source(s) of the autophagosomal membrane are still not fully known. The plasma membrane, Golgi, mitochondria, and in particular the ER have all been proposed as membrane sources, and indeed, it is possible that all participate in autophagosome formation, depending upon the nature of the cellular content that is destined for destruction [3].

With regard to the endoplasmic reticulum (ER), attention has focused on the precise ER sub-region that might contribute to autophagosome biogenesis. The ER is a dynamic structure organized into distinct domains, which include rough and smooth ER, flat membranes (sheets), and tubules [4]. This dynamic nature facilitates the interaction of ER membranes with other organelle membranes, such as mitochondria, endosomes, the Golgi, peroxisomes, and the plasma membrane [5]. The regions of ER that form associations with mitochondria are termed mitochondria-associated ER membranes (MAM), and these have been the focus of much recent attention. Up to about 20% of the mitochondrial surface is closely apposed (10- to 30-nm distances) to ER membranes, and these contacts regulate many fundamental physiological processes, including Ca^2+^ homeostasis, phospholipid metabolism, energy metabolism, mitochondrial biogenesis and trafficking, ER stress and the unfolded protein response (UPR), apoptosis, and inflammatory responses [6, 7].

The mechanisms by which regions of ER come into contact with mitochondria to form MAM are not properly understood, but electron microscopy (EM) studies reveal the presence of structures that appear to tether the two organelles [8]. Recently, the integral ER protein vesicle-associated membrane protein-associated protein B (VAPB) was shown to bind to the outer mitochondrial membrane protein, protein tyrosine phosphatase interacting protein 51 (PTPIP51) to form at least some of these tethers [9]. Evidence to demonstrate that VAPB and PTPIP51 are ER-mitochondria tethers comes from multiple experimental approaches. First, VAPB is an ER protein enriched in MAM, and PTPIP51 is a known outer mitochondrial membrane protein [6, 10]. Second, VAPB and PTPIP51 interact in a large number of different biochemical assays [9, 10, 11, 12]. Third, modulating expression of VAPB or PTPIP51 affects Ca^2+^ exchange between the two organelles, which is a physiological readout of ER-mitochondria contacts [9, 10]. Finally, manipulating VAPB and/or PTPIP51 expression induces appropriate changes in ER-mitochondria contacts as assayed in the EM; VAPB or PTPIP51 small interfering RNA (siRNA) knockdown decreases, whereas overexpression markedly increases ER-mitochondria contacts [9, 13]. High-resolution imaging, such as EM, is required to properly quantify ER-mitochondria contacts of 10- to 30-nm distances [6].

Here, we address the role of MAM and the VAPB-PTPIP51 tethers in autophagy. We show that experimental manipulation of VAPB and PTPIP51 expression to increase and decrease ER-mitochondria associations impacts markedly upon both basal and chemically induced autophagy. We also show that these effects of VAPB and PTPIP51 on autophagy are dependent upon their ER-mitochondria tethering functions because artificial tethering of the two organelles rescues autophagy changes induced by loss of the VAPB-PTPIP51 tethers. Finally, we demonstrate that the mechanism by which the VAPB-PTPIP51 tethers regulate autophagy involves their key role in mediating delivery of Ca^2+^ to mitochondria from ER stores. Our findings reveal a new molecular mechanism for regulating autophagy.

## Results

### siRNA Loss of VAPB and PTPIP51 Induces Autophagy

**Figure 1:**
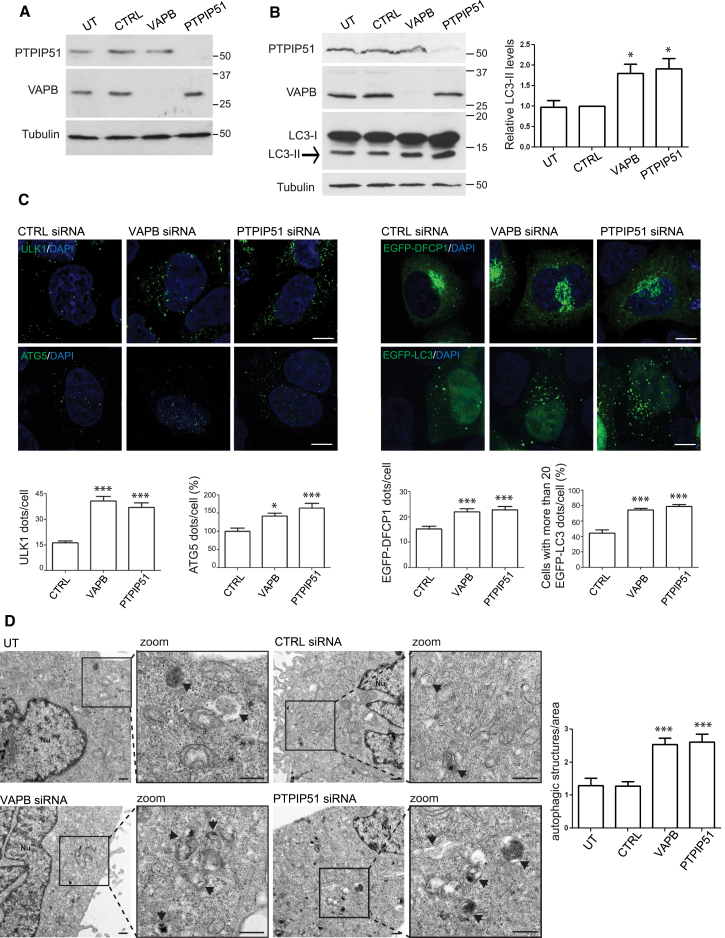
siRNA Knockdown of VAPB or PTPIP51 Increases Autophagic Structures (A) Immunoblots showing siRNA knockdown of VAPB and PTPIP51 in HeLa cells. Cells were untreated (UT) or treated with either control (CTRL), VAPB, or PTPIP51 siRNAs and samples probed for PTPIP51, VAPB, or α-tubulin as a loading control. Protein molecular mass markers are indicated in kD. (B) siRNA knockdown of VAPB or PTPIP51 increases LC3-II levels in HEK293 cells. Cells were UT or treated with either CTRL, VAPB, or PTPIP51 siRNAs and samples probed for PTPIP51, VAPB, LC3, or α-tubulin as a loading control. Both LC3-I and LC3-II isoforms are shown; arrow indicates LC3-II isoform. Bar chart shows relative LC3-II levels following quantification of signals from immunoblots. LC3-II levels were normalized to α-tubulin signals. Protein molecular mass markers are indicated in kD. Data were analyzed by one-way ANOVA and Tukey’s post hoc test; n = 5. Error bars are SEM; ^∗^p ≤ 0.05. (C) siRNA knockdown of VAPB or PTPIP51 increases autophagic structures in HeLa cells. Cells were transfected with CTRL, VAPB, or PTPIP51 siRNAs and the numbers of ULK1, ATG5, EGFP-DFCP1, and EGFP-LC3 autophagic structures quantified. Representative confocal images of cells are shown with DAPI-labeled nuclei; scale bars are 10 μm. Bar charts show quantification of autophagic structures (dots/cell). Data were analyzed by one-way ANOVA and Tukey’s post hoc test. For ULK1, EGFP-DFCP1, and ATG5, n = 45–200 cells; for EGFP-LC3, n = 401–466 cells per condition in five independent experiments. Error bars are SEM; ^∗^p ≤ 0.05; ^∗∗∗^p ≤ 0.001. (D) siRNA knockdown of VAPB or PTPIP51 increases the number of autophagic structures detected in the EM. Representative EM images of UT HeLa cells or cells treated with CTRL, VAPB, or PTPIP51 siRNAs as indicated are shown. Both low- and high-power (zoom) images are displayed. Arrows indicate autophagic structures. The scale bar represents 500 nm. The bar chart shows number of autophagic structures/μm^2^. Data were analyzed by one-way ANOVA and Tukey’s post hoc test. n = 15–17 cells. Error bars are SEM; ^∗∗∗^p ≤ 0.001.

To gain insight into the role of ER-mitochondria associations in autophagy, we first downregulated VAPB or PTPIP51 expression using siRNAs and monitored basal levels of autophagy in HeLa and HEK293 cells. HeLa and HEK293 cells have been used in many studies of autophagy, e.g. [14, 15]. Such siRNA loss of VAPB and PTPIP51 has been shown to markedly reduce ER-mitochondria contacts in a variety of cell types [9, 10]. We used previously characterized siRNAs, and in agreement with earlier studies, these siRNAs led to an approximate 90% reduction in VAPB and PTPIP51 expression in both cell lines (Figures 1A and 1B). To monitor autophagy, we quantified the number of autophagic structures that were present in cells using markers that are recruited to the phagophore and form the autophagosome at different stages. These markers were ULK1, which is one of the earliest proteins recruited to the phagophore; double FYVE-containing protein 1 (DFCP1), which is another early marker of the phagophore; ATG5, which forms a complex with ATG12 to mediate autophagosome elongation; and finally LC3, which is the most commonly used marker for monitoring autophagy and which is present from the later stages of autophagosome formation to the autolysosome [14]. Endogenous ULK1 and ATG5 were detected by immunostaining, whereas DFCP1 and LC3 were detected by transfection of EGFP-tagged proteins. Loss of VAPB or PTPIP51 induced a marked increase in the number of structures labeled by all of these autophagy markers (Figure 1C).

To complement these light microscopy studies, we also monitored how siRNA loss of VAPB or PTPIP51 affected autophagosome numbers as detected by EM. EM has been used in many studies of autophagy [14, 16]. In the EM, autophagic vacuoles are discernible as double-membrane vacuoles containing engulfed cytosolic contents or heterogeneous electron-dense structures with undigested material; these features permit detection and quantification of autophagic vacuoles [14, 16]. EM confirmed that loss of VAPB or PTPIP51 increased the number of autophagic vacuoles (Figure 1D).

**Figure 2:**
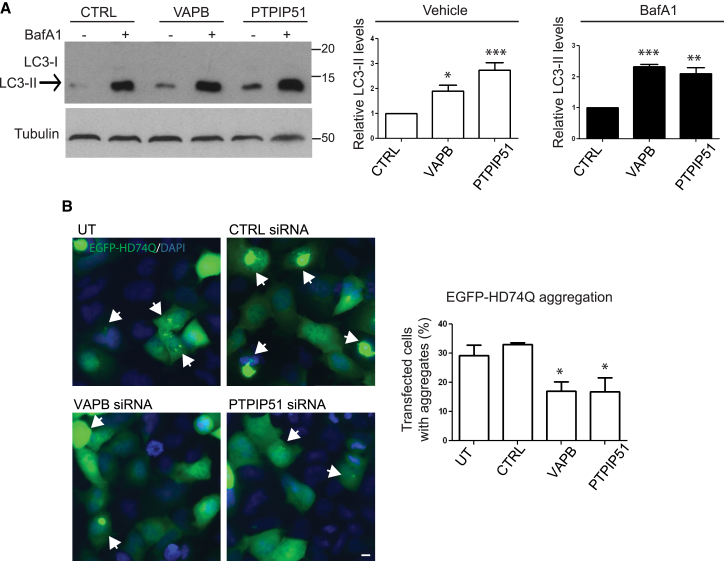
siRNA Knockdown of VAPB or PTPIP51 Induces Autophagic Flux (A) HeLa cells were treated with CTRL VAPB or PTPIP51 siRNAs and treated with either vehicle or bafilomycin A1 (±BafA1) as indicated and samples then probed on immunoblots for LC3 and α-tubulin as a loading control. Both LC3-I and LC3-II isoforms are shown; arrow indicates LC3-II isoform. Bafilomycin A1 increases the levels of LC3-II in control, VAPB, and PTPIP51 siRNA knockdown cells. Bar chart shows relative LC3-II levels following quantification of signals from immunoblots. LC3-II levels were normalized to α-tubulin signals. Protein molecular mass markers are indicated in kD. Data were analyzed by one-way ANOVA and Tukey’s post hoc test; n = 5 (vehicle) and n = 3 (bafilomycin A1). Error bars are SEM; ^∗^p ≤ 0.05; ^∗∗^p ≤ 0.01; ^∗∗∗^p ≤ 0.001. (B) siRNA knockdown of VAPB or PTPIP51 decrease EGFP-HD74Q aggregation. Representative images of HEK293 cells transfected with EGFP-HDQ74 and either UT or treated with CTRL, VAPB, or PTPIP51 siRNAs are shown. Cells were analyzed 48 hr post EGFP-HDQ74 transfection. Arrows indicate cells containing EGFP-HDQ74 aggregates. Blue represents DAPI staining of nuclei. The scale bar represents 10 μm. Bar chart shows percentage of EGFP-HD74Q transfected cells displaying aggregates. Data were obtained from 300–550 EGFP-HD74Q-transfected cells per condition in three independent experiments. Data were analyzed by one-way ANOVA and Tukey’s post hoc test. Error bars are SEM; ^∗^p ≤ 0.05.

The increases in autophagic structures seen following loss of VAPB and PTPIP51 in the above assays could be due to an induction of autophagy or, alternatively, be the consequence of reduced autophagosome turnover. To address this issue, we monitored LC3-II formation by immunoblotting in VAPB and PTPIP51 siRNA knockdown HeLa cells that were also treated with saturating levels of bafilomycin A1. Bafilomycin A1 is an inhibitor of the vacuolar H^+^-ATPase and so inhibits lysosomal acidification and the fusion between autophagosomes and lysosomes so as to block LC3-II degradation; as such, bafilomycin A1 treatment can aid in monitoring autophagosome synthesis [17]. As was the case with HEK293 cells (Figure 1B), siRNA loss of VAPB and PTPIP51 increased the amounts of LC3-II in HeLa cells (Figure 2A). Moreover, LC3-II levels in the presence of bafilomycin A1 increased in control siRNA cells, but this increase was augmented in VAPB and PTPIP51 siRNA knockdown cells, suggesting that loss of VAPB and PTPIP51 stimulates autophagic flux (Figure 2A). To test this further, we monitored aggregation of a widely used model autophagy substrate, EGFP-tagged huntingtin exon 1 containing 74 polyglutamine repeats (EGFP-HDQ74) [18]. In agreement with previous studies, transfected EGFP-HDQ74 formed clearly discernible aggregates in some untreated or control-siRNA-treated cells [18]. However, the numbers of these aggregates were significantly decreased in VAPB and PTPIP51 siRNA knockdown cells (Figure 2B). Loss of VAPB and PTPIP51 therefore appears to enhance clearance of EGFP-HDQ74 aggregates, which is consistent with the effects of VAPB or PTPIP51 loss in the LC3 turnover assays. Collectively, these findings demonstrate that loss of VAPB and PTPIP51 to reduce ER-mitochondria associations stimulates autophagy flux.

### Overexpression of VAPB and PTPIP51 Impairs Autophagy

**Figure 3:**
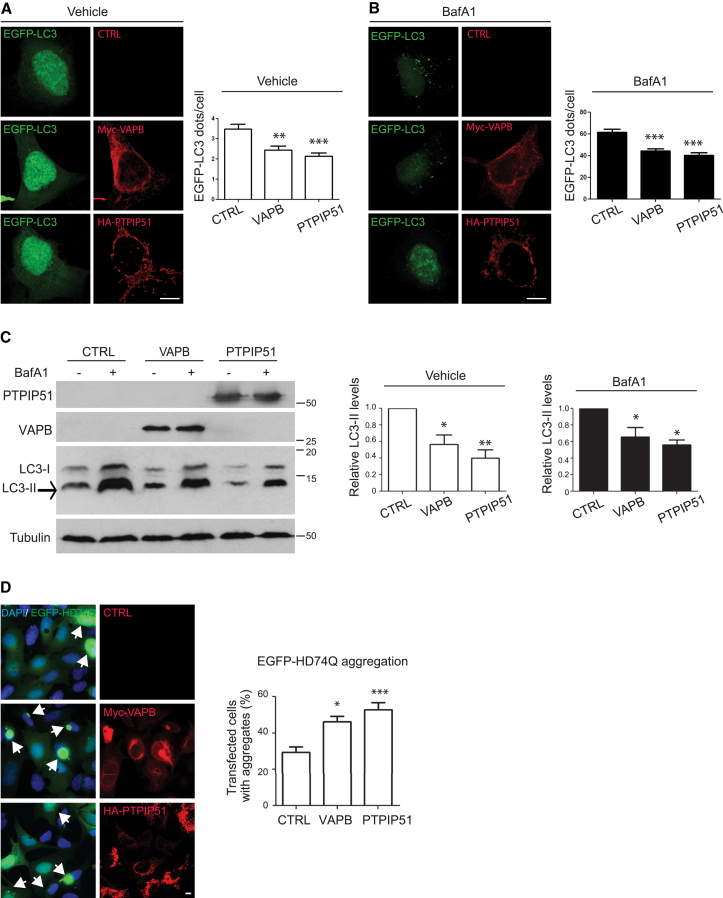
VAPB or PTPIP51 Overexpression Inhibits Basal Autophagy and Autophagic Flux (A and B) Representative images of HEK293 cells co-transfected with EGFP-LC3 and either control empty vector (CTRL), Myc-VAPB, or HA-PTPIP51 and treated with either vehicle (A) or bafilomycin A1 (BafA1) (B) as indicated. Cells were immunostained for VAPB and PTPIP51 via their epitope tags and LC3 visualized via the EGFP tag. Transfection of VAPB or PTPIP51 decreases the numbers of EGFP-LC3 structures in both vehicle- and bafilomycin A1-treated cells. The scale bars represent 10 μm. The bar charts show numbers of EGFP-LC3 dots per cell in the different experiments. Data were analyzed by one-way ANOVA and Tukey’s post hoc test. n = 100–130 cells per condition from three independent experiments. Error bars are SEM; ^∗∗^p ≤ 0.01; ^∗∗∗^p ≤ 0.001. (C) VAPB or PTPIP51 overexpression inhibits autophagic flux. HeLa cells were transfected with either control empty vector (CTRL), Myc-VAPB, or HA-PTPIP51 and treated with either vehicle (−) or bafilomycin A1 (BafA1+) as indicated. Samples were then probed on immunoblots for LC3 and α-tubulin as a loading control. Protein molecular mass markers are indicated in kD. Both LC3-I and LC3-II isoforms are shown; arrow indicates LC3-II isoform. Also shown are immunoblots for PTPIP51 and VAPB, which were detected via their epitope tags. VAPB and PTPIP51 expression decreases the levels of LC3-II in both vehicle- and bafilomycin-A1-treated cells. The bar chart shows relative LC3-II levels following quantification of signals from immunoblots. LC3-II levels were normalized to α-tubulin signals. Data were analyzed by one-way ANOVA and Tukey’s post hoc test; n = 3. Error bars are SEM; ^∗^p ≤ 0.05; ^∗∗^p ≤ 0.01. (D) VAPB and PTPIP51 expression increases EGFP-HD74Q aggregation in HEK293T cells. Cells were co-transfected with EGFP-HD74Q and either control empty vector (CTRL), Myc-VAPB, or HA-PTPIP5 and immunostained for VAPB and PTPIP51 via their epitope tags. Cells were analyzed 48 hr post-transfection. Arrows indicate cells containing EGFP-HDQ74 aggregates; blue represents DAPI staining of nuclei. The scale bar represents 10 μm. The bar chart shows percentage of EGFP-HD74Q-transfected cells displaying aggregates. Data were obtained from 320-580 EGFP-HD74Q-transfected cells per condition from five independent experiments. Data were analyzed by one-way ANOVA and Tukey’s post hoc test. Error bars are SEM; ^∗^p ≤ 0.05; ^∗∗∗^p ≤ 0.001.

We next enquired how increasing ER-mitochondria associations affected autophagy. To do so, we co-transfected cells with either control vector, VAPB, or PTPIP51 and EGFP-LC3 as a marker for autophagic structures. Such overexpression of VAPB or PTPIP51 has been shown to markedly increase ER-mitochondria contacts [9, 13]. Transfection of VAPB or PTPIP51 both decreased the number of EGFP-LC3 autophagic structures in the cells (Figure 3A). Moreover, whereas treatment of the cells with bafilomycin A1 to block LC3 degradation markedly increased the number of EGFP-LC3 structures in cells transfected with control vector, this increase was significantly reduced in the VAPB or PTPIP51 co-transfected cells (Figures 3A and 3B).

To complement these findings, we enquired how overexpression of VAPB or PTPIP51 affected LC3-II levels by immunoblotting. LC3-II levels were reduced in VAPB- and PTPIP51-transfected cells, but whereas treatment with bafilomycin A1 increased the levels of LC3-II in control cells, the magnitude of this increase was reduced in VAPB- and PTPIP51-transfected cells (Figure 3C). These effects of bafilomycin A1 suggest that the overexpression of VAPB and PTPIP51 inhibits autophagosome production.

To test this possibility further, we monitored how overexpression of VAPB or PTPIP51 affected aggregation of EGFP-HDQ74. Cells were co-transfected with EGFP-HDQ74 and either control vector, VAPB, or PTPIP51 and the number of cells with EGFP-HDQ74 aggregates quantified. Co-transfection of VAPB or PTPIP51 both increased the proportion of cells containing EGFP-HDQ74 aggregates, which is again consistent with a decrease in autophagy (Figure 3D).

Expression of EGFP-HDQ74 is toxic to cells, and autophagic clearance of EGFP-HDQ74 is protective against this toxicity [18]. We therefore investigated how modulating expression of VAPB and PTPIP51 affected EGFP-HDQ74 toxicity by monitoring DAPI-stained nuclear morphology. Fragmented or pyknotic nuclei have been shown to be specific markers for cell death in EGFP-HDQ74-expressing cells, which shows a very high correlation with propidium iodide staining of live cells [18]. Compared to control vector, overexpression of VAPB or PTPIP51 both significantly increased the number of EGFP-HDQ74-transfected cells displaying abnormal nuclei (EGFP-HDQ74+control 8.1 ± 1.8, EGFP-HDQ74+VAPB 17.3 ± 2.0 abnormal nuclei, p ≤ 0.01, Student’s t test; EGFP-HDQ74+control 8.1 ± 1.8, EGFP-HDQ74+PTPIP51 21.2 ± 6.4 abnormal nuclei, p ≤ 0.05, Student’s t test; n = 468–610 cells). By contrast, siRNA loss of PTPIP51 reduced the numbers of EGFP-HDQ74 cells displaying abnormal nuclei (EGFP-HDQ74+control 11.0 ± 1.2; EGFP-HDQ74+PTPIP51 siRNA 4.7 ± 0.6 abnormal nuclei; p ≤ 0.05; Student’s t test; n = 361–440 cells). siRNA loss of VAPB showed a trend toward reducing the number of EGFP-HDQ74 cells displaying abnormal nuclei, but this did not reach significance. However, caution must be taken in interpreting such results as being solely linked to EGFP-HDQ74 toxicity. Tightening and loosening of ER-mitochondria contacts for extended periods are both predicted to be detrimental to cells. Tightening can induce Ca^2+^ overload in mitochondria, which leads to opening of the mitochondrial permeability transition pore and signaling for apoptosis; loosening reduces the ability of mitochondria to generate ATP [6, 7]. Such effects may contribute to cellular toxicity via routes that are independent of EGFP-HDQ74 autophagic clearance.

### Overexpression of VAPB and PTPIP51 Impairs Rapamycin- and Torin-1-Induced, but Not Starvation-Induced, Autophagy

**Figure 4:**
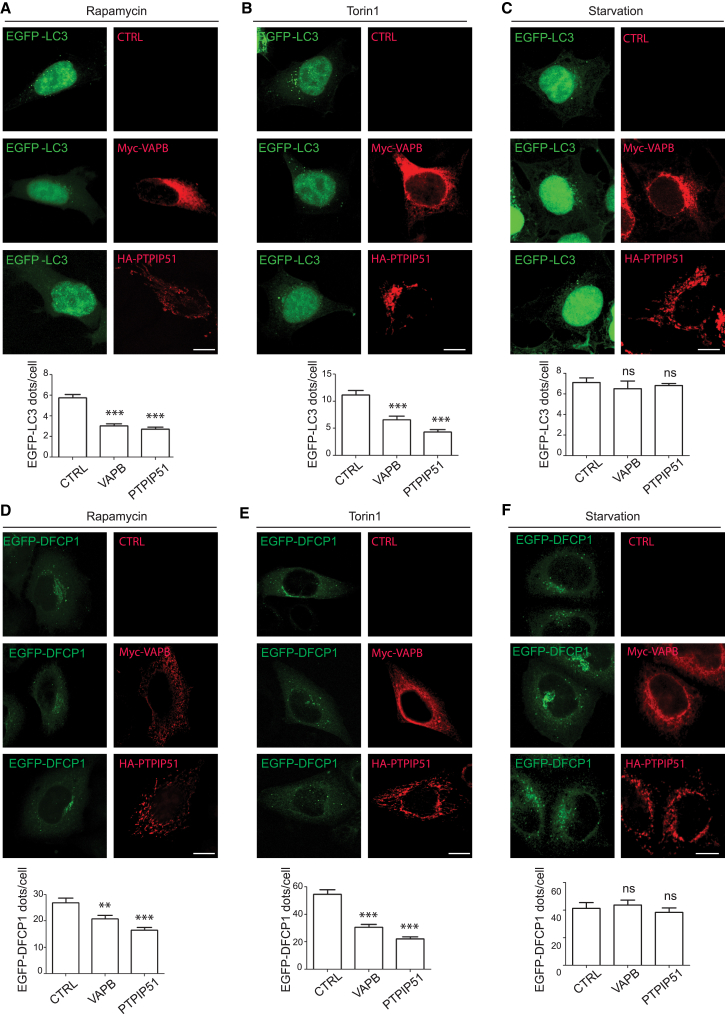
VAPB and PTPIP51 Overexpression Inhibits Rapamycin- and Torin-1-Induced, but Not Starvation-Induced, Autophagy (A–C) Representative images of HEK293T cells co-transfected with EGFP-LC3 and either control empty vector (CTRL), Myc-VAPB, or HA-PTPIP51 as indicated and treated with rapamycin (A), torin 1 (B), or starvation (C). Cells were immunostained for VAPB and PTPIP51 via their epitope tags and LC3 visualized via the EGFP tag. The bar charts show number of EGFP-LC3 dots per cell in the different experiments. Data were analyzed by one-way ANOVA and Tukey’s post hoc test. n = 80–192 cells per condition from three independent experiments. Error bars are SEM; ^∗∗∗^p ≤ 0.001; ns, not significant. (D–F) Representative images of HeLa cells co-transfected with EGFP-DFCP1 and either control empty vector (CTRL), Myc-VAPB, or HA-PTPIP51 and treated with rapamycin (D), torin 1 (E), or starvation (F). Cells were immunostained for VAPB and PTPIP51 via their epitope tags and DFCP1 visualized via the EGFP tag. The bar charts show number of EGFP-DFCP1 dots per cell in the different experiments. Data were analyzed by one-way ANOVA and Tukey’s post hoc test. n = 75–100 cells per condition from three independent experiments. Error bars are SEM; ^∗∗^p ≤ 0.01; ^∗∗∗^p ≤ 0.001. Scale bars represent 10 μm. See also Figure S2.

The above studies show that overexpression of VAPB or PTPIP51 to increase ER-mitochondria associations leads to a reduction in basal autophagy. To determine the effects of VAPB and PTPIP51 overexpression on chemically induced autophagy, we quantified the numbers of EGFP-LC3 and EGFP-DFCP1 autophagic structures in co-transfected cells with either control vector, VAPB, or PTPIP51 and treated with vehicle, rapamycin, or torin 1. Rapamycin and torin 1 are two structurally distinct compounds that induce autophagy by inhibiting the mammalian target of rapamycin (mTOR); mTOR negatively regulates autophagy [19]. As expected, rapamycin and torin 1 markedly increased the numbers of EGFP-LC3 structures in control-transfected cells (rapamycin: numbers of structures in vehicle-treated cells 3.6 ± 0.4, number of structures in rapamycin-treated cells 5.7 ± 1.2, p ≤ 0.05, Student’s t test; torin A: number of structures in vehicle-treated cells 3.9 ± 0.6, number of structures in torin 1-treated cells 12.7 ± 3.1, p ≤ 0.01, Student’s t test; 80–100 cells were analyzed per condition from three independent experiments). However, these effects of rapamycin and torin 1 were significantly reduced in cells co-transfected with either VAPB or PTPIP51 (Figures 4A and 4B). Likewise, rapamycin and torin 1 markedly increased the numbers of EGFP-DFCP1 structures in control-transfected cells (rapamycin: numbers of structures in vehicle-treated cells 17.7 ± 9.6, number of structures in rapamycin-treated cells 26.9 ± 15.7, p ≤ 0.05, Student’s t test; torin A: number of structures in vehicle-treated cells 17.7 ± 9.6, number of structures in torin 1-treated cells 60.4 ± 29.3, p ≤ 0.01, Student’s t test; 75–100 cells were analyzed per condition from three independent experiments). Again, these effects of rapamycin and torin 1 were significantly reduced in cells co-transfected with either VAPB or PTPIP51 (Figures 4D and 4E). Thus, consistent with the effects of VAPB and PTPIP51 on basal autophagy, overexpression of VAPB and PTPIP51 both led to a reduction in the number of EGFP-LC3 and EGFP-DFCP1 autophagic structures in cells undergoing chemically induced autophagy.

We also monitored how starvation-induced autophagy was affected by overexpression of VAPB or PTPIP51. To do so, we grew cells in media lacking all amino acids, as described by others [15, 20, 21]. As expected, starvation significantly increased the numbers of both EGFP-LC3 and EGFP-DFCP1 structures in transfected HEK293 cells (numbers of EGFP-LC3 in control cells 3.6 ± 2.5, number of EGFP-LC3 structures in starved cells 9.2 ± 6, p ≤ 0.05, Student’s t test; numbers of EGFP-DFCP1 in control cells 23 ± 12.8, number of EGFP-DFCP1 structures in starved cells 41.3 ± 36.2, p ≤ 0.001, Student’s t test; 74 to 75 cells were analyzed per condition from three independent experiments). However, unlike autophagy induced by rapamycin or torin 1, expression of VAPB or PTPIP51 had no effect on the number of EGFP-LC3 or EGFP-DFCP1 autophagic structures in cells undergoing starvation-induced autophagy (Figures 4C and 4F).

### The Effects of siRNA Loss of VAPB and PTPIP51 on Autophagy Are Rescued by Artificial Tethering of ER and Mitochondria

The above studies demonstrate that VAPB and PTPIP51 regulate autophagy. However, they do not eliminate the possibility that the effects of VAPB and PTPIP51 on autophagy are unrelated to their ER-mitochondria tethering function and are due to some other, as yet uncharacterized, function of these proteins. We therefore monitored autophagosome formation in VAPB or PTPIP51 siRNA knockdown cells, in which breaking of ER-mitochondria contacts was rescued by use of an artificial tether. This tether comprises the outer-mitochondrial-membrane-targeting sequence of mitochondrial A-kinase anchor protein-1 and the ER-targeting sequence of yeast ubiquitin-conjugating enzyme E2 6, fused to the N and C termini, respectively, of red fluorescent protein (Mito-RFP-ER). Expression of Mito-RFP-ER has been shown to artificially increase ER-mitochondria tethering via these targeting sequences, and RFP permits identification of transfected cells [8].

To first confirm that Mito-RFP-ER increases ER-mitochondria tethering in our hands, we monitored its effect on ER-mitochondria associations using proximity ligation assays. For these, we utilized antibodies to the inositol 1,4,5-trisphosphate (IP3) receptor3 and the voltage-dependent anion channel (VDAC1) because IP3 receptors located in MAM form a functional connection with the outer mitochondrial membrane protein VDAC1 to facilitate Ca^2+^ exchange between the two organelles [6, 7]. The distances detected by proximity ligation assays are similar to those detected by resonance energy transfer between fluorophores (i.e., approximately 10 nm) [22], and so these assays are suitable for detection of ER-mitochondria contacts. Indeed, proximity ligation assays, including ones for the IP3 receptor and VDAC1, have already been used to quantify ER-mitochondria associations [10, 12, 23].

To demonstrate the specificity of the proximity ligation assays, we first performed control experiments in which primary IP3 receptor3 and/or VDAC1 antibodies were omitted. Omission of primary antibodies produced none or only very few signals, whereas inclusion of both antibodies generated large numbers of signals (IP3 receptor3 antibody only: 0.209 ± 0.048 signals/cell, n = 105 cells; VDAC1 antibody only: 0.085 ± 0.029 signals/cell, n = 94 cells; IP3 receptor3+VDAC1 antibodies 61.35 ± 5.43 signals/cell, n = 55 cells; from three independent experiments). As expected, siRNA loss of VAPB or PTPIP51 decreased whereas expression of Mito-RFP-ER increased IP3 receptor3-VDAC1 proximity ligation assay signals in cells (Figure S1). Moreover, expression of Mito-RFP-ER partially rescued the effects of loss of VAPB or PTPIP51 on these signals (Figure S1).

**Figure 5:**
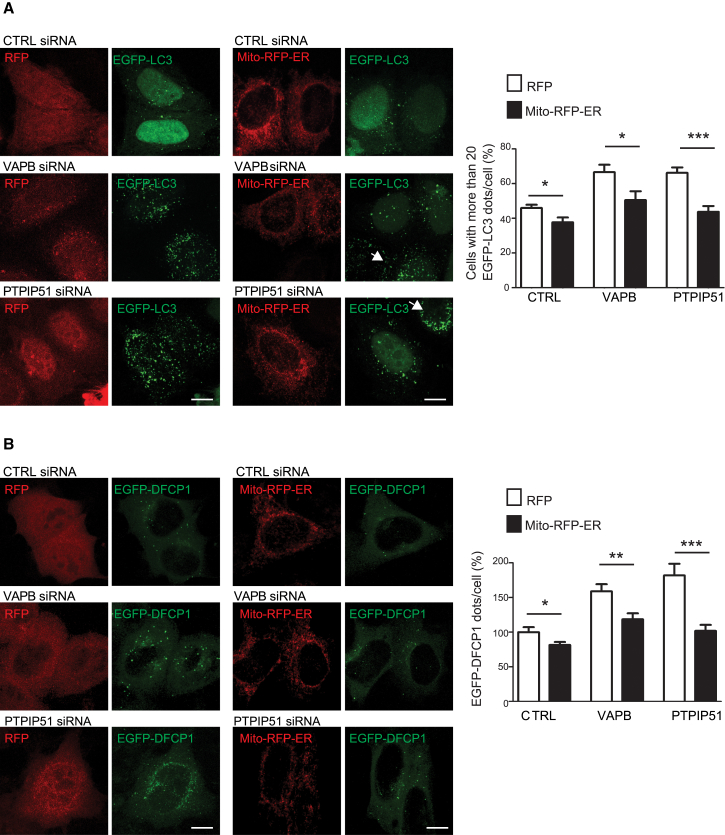
Artificially Tethering ER and Mitochondria by Transfection of Mito-RFP-ER Reduces the Effect of siRNA Loss of VAPB or PTPIP51 on Autophagosome Formation (A) Representative images of HEK293 cells treated with either control, VAPB, or PTPIP51 siRNAs and then transfected with EGFP-LC3+control RFP or EGFP-LC3+Mito-RFP-ER as indicated; arrows show non-transfected cells for comparison. The scale bars represent 10 μm. (B) Representative images of HeLa cells treated with either control, VAPB, or PTPIP51 siRNAs and then transfected with EGFP-DFCP1+RFP or EGFP-DFCP1+Mito-RFP-ER as indicated. The scale bars represent 10 μm. The bar charts show quantification of EGFP-LC3 (A) and EGFP-DFCP1 (B) autophagic structures (dots/cell). Data were analyzed by Student’s t test. n = 71–264 cells per condition in three to five independent experiments. Error bars are SEM; ^∗^p ≤ 0.05; ^∗∗^p ≤ 0.01; ^∗∗∗^p ≤ 0.001. See also Figure S1.

We therefore monitored how expression of Mito-RFP-ER affected autophagosome formation in VAPB or PTPIP51 siRNA knockdown cells. To do so, we quantified the number of EGFP-LC3 and EGFP-DFCP1 structures in HeLa cells as described earlier (see Figure 1C). Compared to RFP control, expression of Mito-RFP-ER alone decreased the number of EGFP-LC3 and EGFP-DFCP1 structures, which demonstrates further that increasing ER-mitochondria contacts inhibits autophagosome formation (Figures 5A and 5B). However, whereas loss of VAPB or PTPIP51 increased the numbers of EGFP-LC3 and EGFP-DFCP1 structures (in agreement with earlier studies; Figure 1C), these increases were significantly reduced in cells co-transfected with Mito-RFP-ER (Figures 5A and 5B). Thus, the increases in autophagosome formation induced by loss of VAPB or PTPIP51 expression are linked to the ER-mitochondria tethering function of VAPB and PTPIP51.

We also monitored how expression of the Mito-RFP-ER artificial tether affected chemically and starvation-induced autophagy. To do so, we monitored the numbers of EGFP-LC3 structures in torin 1 and starved HEK293 cells that were also transfected with Mito-RFP-ER. In agreement with studies on the effects of the VAPB-PTPIP51 tethers on chemically and starvation-induced autophagy (Figure 4), expression of Mito-RFP-ER reduced the numbers of EGFP-LC3 autophagic structures in torin 1, but not starved cells (Figure S2).

### The Effect of VAPB and PTPIP51 on Autophagy Involves Their Role in Mediating ER-Mitochondria Ca^2+^ Exchange

A primary function of ER-mitochondria contacts is to facilitate delivery of Ca^2+^ to mitochondria from ER stores. A major route for this delivery involves release of Ca^2+^ from IP3 receptors located in MAM for uptake by mitochondria via VDAC and the mitochondrial Ca^2+^ uniporter [6, 7]. The tight contacts between ER and mitochondria at MAM facilitate this exchange because they enable high local concentrations of Ca^2+^ (Ca^2+^ puffs) to be achieved, which are sufficient to drive a response at the mitochondrial surface [6, 7].

**Figure 6:**
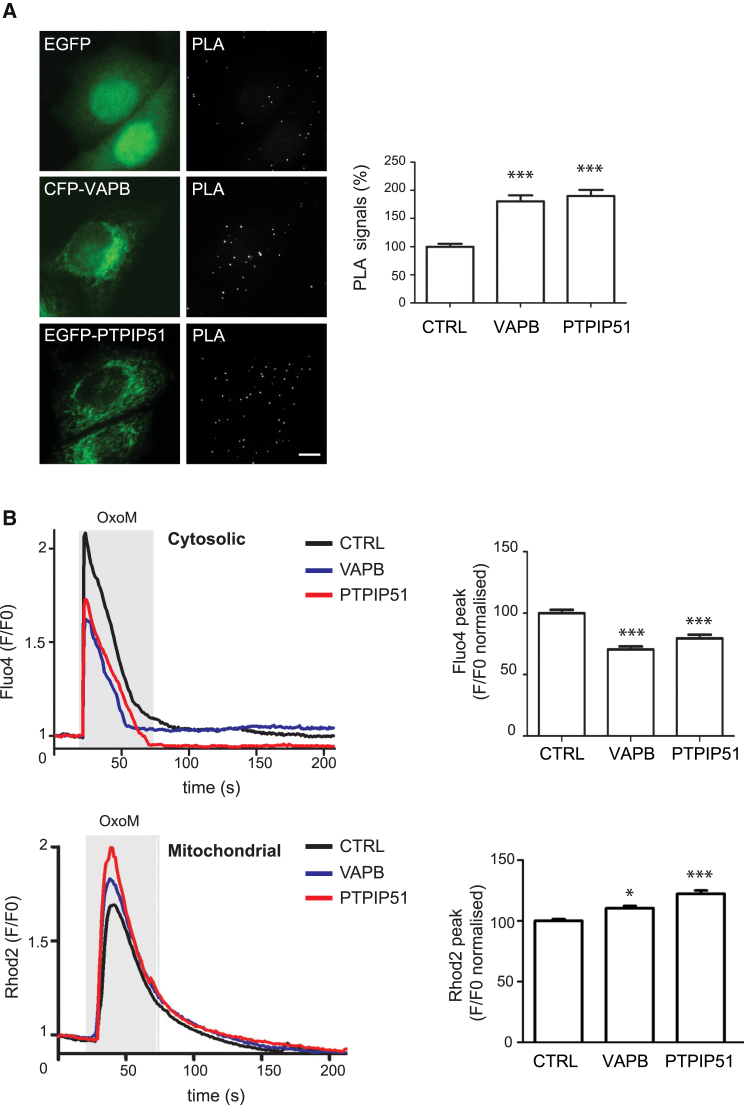
Overexpression of VAPB or PTPIP51 Increases IP3 Receptor3-VDAC1 Interactions and ER-Mitochondria Ca2+ Exchange (A) HeLa cells were transfected with control EGFP vector, CFP-VAPB, or EGFP-PTPIP51 and proximity ligation assays (PLAs) for IP3 receptor3-VDAC1 interactions then performed. Representative images with PLA signals in the different transfected cells are shown. The scale bar represents 10 μm. The bar chart shows quantification of PLA signals. Data were analyzed by one-way ANOVA and Tukey’s post hoc test. n = 77–142 cells per condition from three independent experiments. Error bars are SEM; ^∗∗∗^p ≤ 0.001. (B) Cytosolic (upper) and mitochondrial (lower) Ca^2+^ levels following oxotremorine-M (OxoM)-induced Ca^2+^ release from ER stores. HEK293 cells were co-transfected with M3R and either control empty vector (CTRL), Myc-VAPB, or HA-PTPIP51 and treated with oxotremorine-M. Representative traces of Fluo4 (cytosolic) and Rhod2 (mitochondrial) fluorescence are shown on the left, and normalized peak values are shown on the right. Fluo4 and Rhod2 fluorescence shows transient increases in cytosolic and mitochondrial Ca^2+^ levels upon OxoM-induced Ca^2+^ release from ER stores. Compared to control, VAPB and PTPIP51 expression decreased peak cytosolic and increased peak mitochondrial Ca^2+^ levels. Data were analyzed by one-way ANOVA and Tukey’s post hoc test. n = 48–81 cells from three independent experiments. Error bars are SEM; ^∗^p ≤ 0.05; ^∗∗∗^p ≤ 0.001. See also Figure S3.

A number of studies have now shown that reductions in IP3 receptor function and impaired Ca^2+^ uptake by mitochondria stimulate autophagy [24, 25, 26, 27, 28, 29]. Thus, the effects of VAPB and PTPIP51 on autophagy may be linked to Ca^2+^ exchange via MAM at ER-mitochondria contact sites. To test this possibility further, we first monitored whether siRNA knockdown or overexpression of VAPB or PTPIP51 to decrease or increase ER-mitochondria contacts, respectively, affected expression of key ER-mitochondria Ca^2+^ exchange proteins. To do so, we probed immunoblots of VAPB/PTPIP51-transfected/siRNA-treated HEK293 cells for IP3 receptor, VDAC, or the mitochondrial Ca^2+^ uniporter. However, we detected no changes in expression of any of these proteins in the VAPB or PTPIP51 siRNA knockdown or transfected cells (Figures S3A and S3B). We next monitored whether overexpression of VAPB or PTPIP51 affected IP3 receptor3-VDAC1 interactions using proximity ligation assays. As predicted, expression of VAPB or PTPIP51 both led to increases in IP3 receptor3-VDAC1 interactions (Figure 6A). These results complement the effects of siRNA knockdown of VAPB or PTPIP51, which induce reductions in IP3 receptor3-VDAC1 interactions (Figure S1). Finally, we monitored how overexpression of VAPB or PTPIP51 affected uptake of Ca^2+^ by mitochondria following IP3 receptor-mediated release from ER stores. For these experiments, we used HEK293 cells co-transfected with the M3 muscarinic-acetylcholine-receptor (M3R) and triggered physiological IP3 receptor-mediated Ca^2+^ release by application of the M3R agonist oxotremorine-M. In line with previous studies on ER-mitochondria associations, we used HEK293 cells for these experiments because they do not express endogenous M3R and so provide a useful model for monitoring mitochondrial Ca^2+^ levels following its release from ER specifically in transfected cells [9, 10, 12]. Transfection of VAPB or PTPIP51 both induced significant increases in mitochondrial and corresponding decreases in cytosolic Ca^2+^ levels in these experiments (Figure 6B). Again, these results complement previous studies that show that siRNA knockdown of VAPB or PTPIP51 reduce mitochondrial and increase cytosolic Ca^2+^ levels following IP3-receptor-mediated Ca^2+^ release [10]. Thus, overexpression and siRNA knockdown of VAPB and PTPIP51 to modulate ER-mitochondria contacts induce appropriate changes in IP3 receptor3-VDAC1 interactions and IP3-receptor-mediated mitochondrial Ca^2+^ uptake. Moreover, these changes do not involve alterations to the levels of expression of IP3 receptor, VDAC, or the mitochondrial Ca^2+^ uniporter.

**Figure 7:**
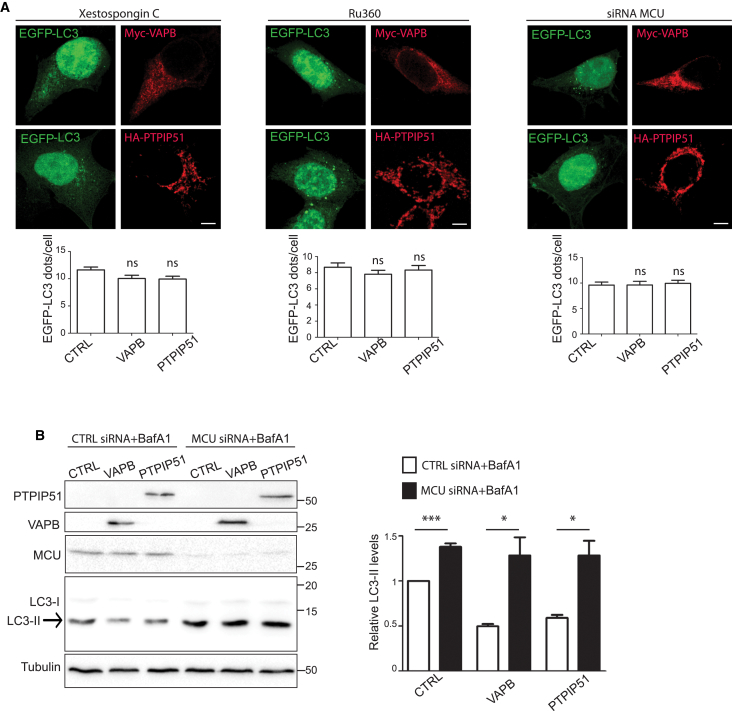
Inhibiting IP3 Receptor-Mediated Ca2+ Delivery to Mitochondria Abrogates the Effects of VAPB and PTPIP51 Overexpression on Autophagosome Formation (A) Representative images of HEK293 cells co-transfected with EGFP-LC3 and either myc-VAPB or HA-PTPIP51 and treated with either the IP3 receptor inhibitor Xestospongin C, the mitochondrial Ca^2+^ uniporter (MCU) blocker Ruthenium-360 (Ru360), or MCU siRNAs. For MCU siRNAs, cells were first treated with siRNAs and then transfected with plasmids. Cells were immunostained for VAPB and PTPIP51 via their epitope tags and LC3 visualized via the EGFP tag. The scale bars represent 10 μm. Bar charts show numbers of EGFP-LC3 dots per cell in the different experiments. Data were analyzed by one-way ANOVA. n = 118–187 cells in three or four independent experiments. Error bars are SEM. (B) siRNA loss of MCU increases LC3-II levels and abrogates the effects of VAPB and PTPIP51 overexpression on LC3-II levels in bafilomycin-A1-treated HeLa cells. Cells were treated with CTRL or MCU siRNAs, transfected with either myc-VAPB or HA-PTPIP51, and treated with bafilomycin A1 (BafA1) as indicated. Samples were then probed on immunoblots for LC3, MCU, VAPB, PTPIP51, and α-tubulin as a loading control. VAPB and PTPIP51 were detected via their epitope tags. Both LC3-I and LC3-II isoforms are shown; arrow indicates LC3-II isoform. Molecular mass markers are indicated in kD. The bar chart shows relative LC3-II levels following quantification of signals from immunoblots. LC3-II levels were normalized to α-tubulin signals. Data were analyzed by one-way ANOVA and Tukey’s post hoc test; n = 3. Error bars are SEM; ^∗^p ≤ 0.05; ^∗∗∗^p ≤ 0.001. See also Figure S4.

We therefore enquired whether the reductions in autophagosome formation induced by overexpression of VAPB or PTPIP51 (Figure 3) involve stimulation of Ca^2+^ delivery to mitochondria from IP3 receptors. To do so, we quantified the numbers of EGFP-LC3 autophagic structures in cells co-transfected with control vector, VAPB, or PTPIP51 and then treated with either vehicle, Xestospongin C, or Ruthenium-360 to block ER-mitochondria Ca^2+^ exchange. Xestospongin C is a potent membrane-permeable IP3 receptor antagonist, and Ruthenium-360 is an inhibitor of the mitochondrial Ca^2+^ uniporter [28, 30]. To complement these pharmacological studies, we also monitored how siRNA loss of the mitochondrial Ca^2+^ uniporter affected the number of EGFP-LC3 autophagic structures in the VAPB- or PTPIP51-overexpressing cells. This siRNA treatment induced an approximate 90% reduction in expression of the mitochondrial Ca^2+^ uniporter (Figure S4A). Treatment with Xestospongin C, Ruthenium-360, or mitochondrial Ca^2+^ uniporter siRNAs all induced a significant increase in EGFP-LC3 autophagic structures (Figure S4B). These results are in line with previous studies that also show that inhibiting IP3-receptor-mediated delivery of Ca^2+^ to mitochondria stimulates autophagosome formation [24, 26, 27, 28, 31, 32, 33]. However, the inhibitory effects of VAPB and PTPIP51 overexpression on EGFP-LC3 autophagosome formation (Figure 3) were abrogated in cells treated with Xestospongin C or Ruthenium-360 and in the mitochondrial Ca^2+^ uniporter siRNA knockdown cells (Figure 7A). Moreover, treatment of the cells with bafilomycin A1 to block LC3 degradation not only confirmed that siRNA loss of the mitochondrial Ca^2+^ uniporter increased LC3-II levels, demonstrating an induction of autophagy, but also revealed that this increase was unaffected by overexpression of VAPB or PTPIP51 (Figure 7B). Thus, the inhibitory effects of VAPB and PTPIP51 overexpression on autophagy induction are completely abrogated by blocking IP3-receptor-mediated delivery of Ca^2+^ to mitochondria.

## Discussion

Tight contacts between regions of ER and mitochondria regulate a number of fundamental physiological processes [6, 7]. One mechanism by which these contacts form involves the tethering proteins VAPB and PTPIP51 [9, 10, 13]. Here, we demonstrate that the VAPB-PTPIP51 tethers also regulate autophagosome formation. We show that loosening ER-mitochondria contacts via loss of VAPB or PTPIP51 induces whereas tightening contacts by overexpression of VAPB or PTPIP51 impairs basal autophagy. We also show that chemical induction of autophagy by treatment with rapamycin or torin 1 is impaired in VAPB- or PTPIP51-overexpressing cells. Moreover, we show that expression of a synthetic linker protein that artificially tethers ER and mitochondria also reduces autophagosome formation and that this artificial tether rescues the effects of siRNA loss of VAPB or PTPIP51 on autophagy. Thus, these effects of VAPB and PTPIP51 manipulation on autophagy are associated with their ER-mitochondria tethering function and not some as yet unknown alternative function of these proteins.

Recently, others have also reported a role for ER-mitochondria associations in autophagy and mitophagy [15, 20, 21, 34, 35]. Our findings provide novel mechanistic data to demonstrate that ER-mitochondria contacts mediated by the VAPB-PTPIP51 tethers regulate autophagy. There are, however, some differences between these various studies. Notably, we find that loosening of ER-mitochondria contacts induces autophagosome formation, whereas some others report that such loosening is inhibitory to their formation [15, 20, 21]. The reasons for these dissimilar results may involve the different experimental approaches utilized. For example, in our study, we monitored basal and rapamycin-, torin 1-, and starvation-induced autophagy, whereas others have focused solely on autophagy induced by starvation [15, 20, 21]. Indeed, we found differences in how autophagy induced by rapamycin/torin 1 and starvation were affected by ER-mitochondria tethering.

Additionally, different studies have used different methods to experimentally manipulate ER-mitochondria contacts. In our study, this involved the VAPB-PTPIP51 tethers, but other investigations manipulated mitofusin-2 and phosphofurin acidic cluster sorting protein-2 (PACS-2) [15, 20, 21]. Both mitofusin-2 and PACS-2 have also been linked to ER-mitochondria tethering [36, 37, 38]. One possibility is that different tethering proteins mediate recruitment of distinct ER domains to mitochondria (rough or smooth ER, tubules, or sheets), and these may function to regulate autophagosome formation in different ways, with the precise outcome varying according to the autophagic stimulus and the nature of the structure targeted for autophagic clearance.

Setting aside these possibilities, the roles of mitofusin-2 and PACS-2 as ER-mitochondria tethering proteins are unclear. Thus, whereas some reports show that loss of mitofusin-2 reduces ER-mitochondria contacts [36, 38], other studies contradict these findings and claim that such loss increases ER-mitochondria contacts [39, 40, 41, 42, 43]. Clearly, if mitofusin-2 loss increases rather than decreases ER-mitochondria tethering, then prior experiments involving loss of mitofusin-2 can be reinterpreted as showing that increased ER-mitochondria associations reduce autophagy.

Likewise, the precise role of PACS-2 in regulating ER-mitochondria associations is not properly established. PACS-2 is a multifunctional sorting protein involved in inter-organelle trafficking in the secretory and endosomal pathways [44]. In addition, it also translocates to the nucleus to regulate SIRT1-mediated deacetylation of p53 [45]. How these diverse functions link to ER-mitochondria associations are unclear, and certainly, there is no direct evidence to suggest that PACS-2 acts as an ER-mitochondria tethering protein, such as VAPB or PTPIP51. Rather, PACS-2 may function to somehow indirectly influence or regulate ER-mitochondria associations. Additionally, PACS-2 impacts upon mitochondrial morphology with loss of PACS-2 inducing extensive mitochondrial fragmentation [37]. The diversity of cell functions regulated by PACS-2 and these caveats involving mitochondrial morphology query the validity of concluding that changes in autophagosome formation induced by loss of PACS-2 are due solely to disruption of ER-mitochondria contacts.

Here, we investigated how altering ER-mitochondria associations via manipulating VAPB and PTPIP51 affects autophagy. As detailed above (see Introduction), a number of lines of evidence demonstrate that VAPB and PTPIP51 function as ER-mitochondria tethers [9, 10, 11, 12, 13]. We also utilized an artificial tether to manipulate ER-mitochondria associations, and this produced complementary results to those involving VAPB and PTPIP51. Thus, our approach provides a more direct and easily interpretable route for probing the role of ER-mitochondria contacts in autophagosome formation.

Our findings are also in line with a large number of studies that show that disruption of Ca^2+^ transfer from MAM-located IP3 receptors to mitochondria stimulates autophagy [24, 25, 26, 27, 28, 29, 31, 32]. This, stimulation is proposed to represent a physiological response of the cell to altered bioenergetics because mitochondria require Ca^2+^ for efficient production of ATP (several dehydrogenases in the tricarboxylic acid cycle are Ca^2+^ regulated) [6, 7, 46]. A primary function of ER-mitochondria contacts is to facilitate IP3-receptor-mediated delivery of Ca^2+^ to mitochondria [6, 7, 8]. Consistent with these roles, loss of VAPB or PTPIP51 reduces whereas overexpression of VAPB or PTPIP51 increases mitochondrial Ca^2+^ uptake following its release from IP3 receptors (Figure 6B) [9, 10]. Together, these findings suggest that the effects of VAPB and PTPIP51 on autophagy are linked to their role in mediating Ca^2+^ exchange between ER and mitochondria at contact sites. In support of this, we found that the effects of VAPB and PTPIP51 overexpression on autophagosome formation were completely abrogated in cells treated with Xestospongin C, Ruthenium-360, or siRNAs for the mitochondrial Ca^2+^ uniporter, which all inhibit IP3-receptor-mediated delivery of Ca^2+^ to mitochondria. Thus, the mechanism by which the VAPB-PTPIP51 tethering proteins impact upon autophagy involves their regulation of ER-mitochondria Ca^2+^ exchange at MAM.

Of interest was our finding that increased ER-mitochondria tethering induced by overexpression of VAPB, PTPIP51, or the Mito-RFP-ER artificial tethers inhibited rapamycin- and torin 1-induced, but not starvation-induced, autophagy. Rapamycin and torin 1 induce autophagy by selectively inhibiting mTOR [19]. By contrast, starvation-induced autophagy is more complex, involving mTOR and a variety of upstream nutrient-sensing molecules, including AMP-activated protein kinase and Ca^2+^-calmodulin-dependent protein kinase β [46]. Starvation-induced, but not rapamycin-induced, autophagy is therefore dependent upon disruption of the BCL2-Beclin 1 interaction [47]. Also, whereas numerous studies have shown that blocking IP3-receptor-mediated delivery of Ca^2+^ to mitochondria stimulates autophagy [24, 25, 26, 27, 28, 29, 31, 32] (and results shown here), paradoxically, starvation-induced autophagy requires release of Ca^2+^ from sensitized IP3 receptors [48]. Thus, the different effects of overexpression of VAPB, PTPIP51, and the artificial tethers on starvation- and rapamycin/torin 1-induced autophagy are probably due to differences in the signaling mechanism by which these stimuli induce autophagy. Whatever the precise scenario, the results described here demonstrate that the VAPB-PTPIP51 tethers regulate basal and rapamycin- and torin 1-induced autophagy and that this involves their role in facilitating IP3-receptor-mediated delivery of Ca^2+^ from ER stores to mitochondria.

Increasing evidence supports a role for autophagy in neurodegenerative diseases, and damage to ER-mitochondria signaling is a feature of these diseases [2, 6, 49, 50]. It will therefore be interesting to determine whether there are links between neurodegenerative disease insults, the VAPB-PTPIP51 tethers, and autophagy. The results described here form the basis for such studies.

## Experimental Procedures

### Reagents

Details of plasmids, siRNAs, antibodies, and other reagents are described in Supplemental Experimental Procedures.

### Cell Culture, Transfection, SDS-PAGE, and Immunoblotting

Cells were transfected with plasmids, treated with siRNAs, and analyzed by SDS-PAGE and immunoblotting essentially as described [9, 10, 12]. Full details are provided in Supplemental Experimental Procedures.

### Microscopy

Cells were fixed and analyzed by immunofluorescence microscopy and EM essentially as previously described [9, 12]. Proximity ligation assays were performed using a Duolink In Situ Far Red kit according to the manufacturer’s instructions (Sigma). Ca^2+^ measurements were obtained as described previously [9, 10, 12]. Confocal and wide-field images were acquired using Leica TCS-SP5 and Leica DM5000B microscopes, EM images acquired using a Tecnai 20 instrument, and Ca^2+^ measurements obtained with Zeiss Axiovert S100 or Nikon Eclipse TiE microscopes. Full details of microscopy methods and statistical and other analyses are provided in Supplemental Experimental Procedures.

### Statistical Analyses

Statistics were performed using GraphPad Prism.

## Author Contributions

P.G.-S. and C.C.J.M. designed the study. P.G.-S. performed most experiments, analyzed data, and wrote the manuscript. S.P. and R.S. performed Ca^2+^ and other experiments. C.C.J.M., W.N., and D.P.H. designed and supervised experiments and wrote the manuscript. All authors edited the manuscript.

## Supplemental Information

Document S1. Figures S1–S4 and Supplemental Experimental Procedures

Document S2. Article plus Supplemental Information
