# Supplementary material for: The ER-Mitochondria Tethering Complex VAPB-PTPIP51 Regulates Autophagy
Source: Curr Biol. 2017 Feb 6;27(3):371–85. doi: 10.1016/j.cub.2016.12.038 (PMC5300905; doi:10.1016/j.cub.2016.12.038)
Supplement: Document S1. Figures S1–S4 and Supplemental Experimental Procedures [file mmc1.pdf]

Current Biology, Volume *27*

Supplemental Information

The ER-Mitochondria Tethering Complex

VAPB-PTPIP51 Regulates Autophagy

Patricia Gomez-Suaga, Sebastien Paillusson, Radu Stoica, Wendy Noble, Diane P. Hanger, and Christopher C.J. Miller

Supplemental Figures

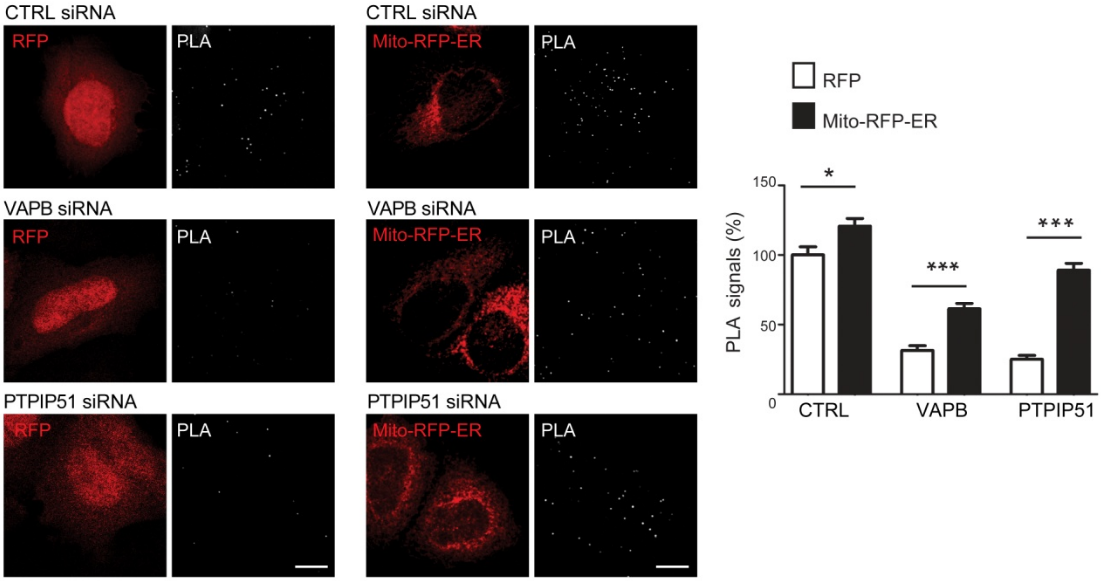

Figure S1 (related to Figure 5). Expression of the artificial ER-mitochondria tethering protein Mito-RFP-ER increases IP3 receptor3-VDAC1 interactions. HeLa cells were treated with either control (CTRL), VAPB or PTPIP51 siRNAs and then transfected with either control RFP (left panel) or Mito-RFP-ER (right panel) plasmids. Proximity ligation assays (PLA) for IP3 receptor3-VDAC1 interactions were then performed. Representative confocal images of the different transfected cells are shown along with PLA signals. Scale bars are 10 μm. Bar chart shows quantification of PLA signals. Data were analysed by Students T test. N=45-160 cells per condition from 3-4 independent experiments. Error bars are s.e.m.; *p≤ 0.05; ***p≤0.001.

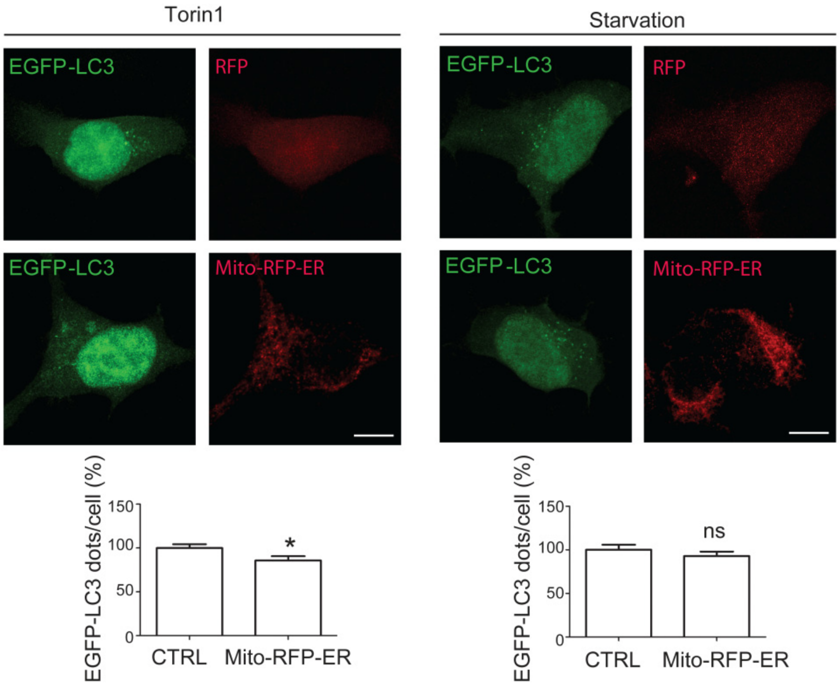

Figure S2 (related to Figure 4). Expression of the artificial ER-mitochondria tethering protein Mito-RFP-ER reduces the numbers of EGFP-LC3 autophagic structures in HEK293 cells undergoing autophagy induced by Torin 1 but not starvation. Representative images of cells co-transfected with EGFP-LC3 and either RFP control of Mito-RFP-ER, and then treated with Torin 1 or starvation as indicated. Scale bars are 10 µm. Bar charts show relative numbers (%) of EGFP-LC3 dots per cell in the different conditions. Data were analysed by Students T test. N=70-107 cells per condition form 3 independent experiments. Error bars are s.e.m.; *$p \leq 0.05$; ns not significant.

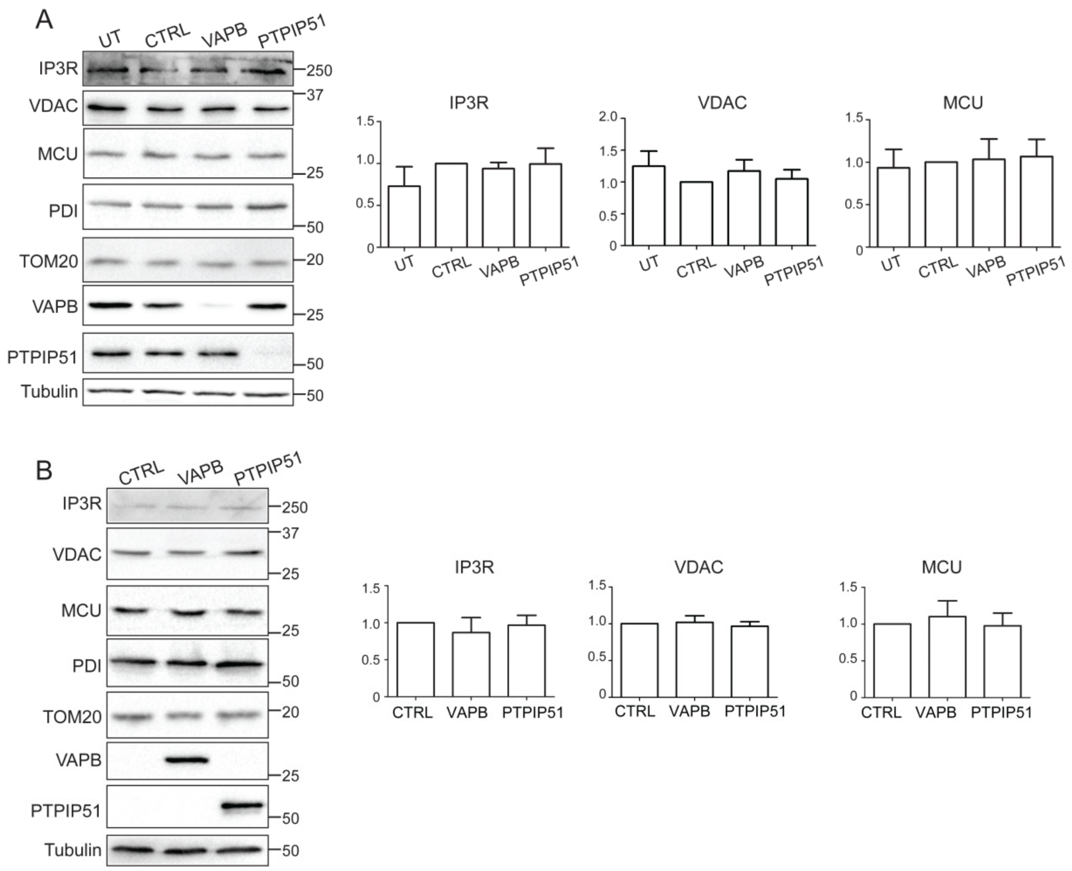

Figure S3 (related to Figure 6). siRNA loss and overexpression by transfection of VAPB or PTPIP51 do not alter expression of the IP3 receptor (IP3R), VDAC or the mitochondrial Ca^{2+} uniporter (MCU). Immunoblots for IP3R, VDAC, MCU, VAPB, PTPIP51 along with loading controls for ER (PDI), mitochondria (TOM20) and α-tubulin are shown. (A) shows cells either untreated (UT) or treated with control, VAPB or PTPIP51 siRNAs. (B) shows cells transfected with either CTRL vector, myc-VAPB or HA-PTPIP51. In (B) VAPB and PTPIP51 were detected via their epitope tags. Molecular mass markers are indicated in kD. Bar charts show relative levels of IP3 receptor, VDAC and MCU after normalization of signals. IP3R was normalized to PDI and VDAC and MCU were normalized to TOM20 signals. Data were analysed by one-way analysis of variance; no significant changes in the levels of IP3R, VDAC or MCU were detected in either the VAPB/PTPIP51 siRNA knockdown or transfected cells compared to controls. N=3-4. Error bars are s.e.m.

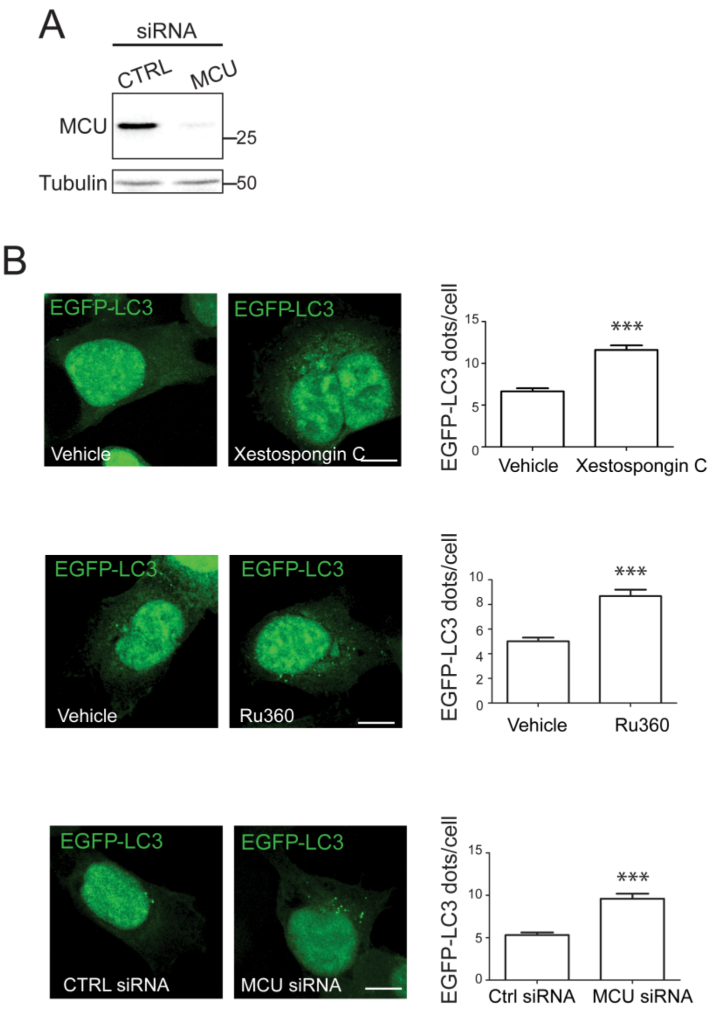

Figure S4, (related to Figure 7). Inhibiting IP3 receptor-mediated Ca^{2+} delivery to mitochondria with Xestospongin C, Ruthenium-360 or siRNA knockdown of the mitochondrial Ca^{2+}uniporter (MCU) stimulates autophagosome formation. (A) siRNA knockdown of MCU. HEK293 cells were treated with control (CTRL) or MCU siRNAs and the samples probed on immunoblots for MCU and α-tubulin as a loading control. (B) Representative images of HEK293 cells transfected with EGFP-LC3 and then treated with either vehicle, Xestospongin C or Ruthenium-360, or treated with control or MCU siRNAs and then transfected with EGFP-LC3. Scale bars are 10 µm. Bar charts show numbers of EGFP-LC3 dots per cell in the different conditions. Data were analysed by Students T test. N=151-188 cells in 3 independent experiments. Error bars are s.e.m; ***$p \leq 0.001$.

Experimental Procedures

Plasmid and siRNAs

Mammalian expression vectors for Myc and CFP-tagged VAPB, hemagglutinin (HA) and EGFP-tagged PTPIP51, and the M3 muscarinic receptor were as described [S1, S2]. pMXs-puro EGFP-DFCP1 was a gift from Noboru Mizushima (Addgene plasmid # 38269), pEGFP-HDQ74 (Addgene plasmid # 40262) and EGFP-LC3 were gifts from David Rubinsztein. The outer mitochondrial membrane-ER linker plasmid (mAKAP1-mRFP-yUBC6; named Mito-RFP-ER here) was a gift from Gyorgy Hajnoczky [S3]. RFP was from Clontech. Human control, VAPB and PTPIP51 siRNAs were as described [S1, S2]. siRNAs for the mitochondrial Ca^{2+} uniporter were an "ON TARGET plus SMART pool" of GAUCAGGCAUUGUGGAAUA, GUUUUGACCUAGAGAAAUA, ACUGAGAGACCCAUUACAA and GUAAUGACACGCCAGGAAU; (siRNA J-015519-17 MCU). All siRNAs were from GE Healthcare Dharmacon.

Antibodies and other reagents

Rabbit antibody to VAPB has been described and was generated by immunization with GST-VAPB (1–220) [S1]. Rabbit anti-PTPIP51 was from Atlas. Rabbit anti-haemagglutin (HA) and mouse anti-α-tubulin (DM1A) were from Sigma. Rabbit anti-LC3, mouse anti-Myc and rabbit anti VDAC were from Cell Signaling Technology. Rabbit anti-ULK1, rabbit anti-translocase of the outer membrane-20 (TOM20) and goat anti VDAC1 were from Santa Cruz Biotechnology. Rabbit anti-ATG5 was from Novus Biologicals. Rabbit anti-IP3 receptor3 was from Millipore. Mouse anti protein disulphide isomerase (PDI) was from Affinity Bioreagents. Secondary antibodies for immunoblotting were horseradish peroxidase-coupled goat anti-mouse and anti-rabbit Igs (GE Healthcare Life Sciences, Piscataway, NJ). Alexa fluorophore (488 and 594)-coupled goat anti-mouse and goat anti-rabbit Igs used for immunofluorescence microscopy were purchased from Invitrogen (Grand Island, NY). Bafilomycin A1 and Xestospongin C were from Sigma; Rapamycin and Torin-1 from were from Biovision, Ruthenium-360 was from Millipore and oxotremorine-M was from Tocris. Oxotremorine-M and Ruthenium-360 were dissolved in water, Rapamycin was dissolved in ethanol and Bafilomycin A1, Torin-1 and Xestospongin C were dissolved in DMSO.

Cell culture and transfection

HeLa and HEK293 cells were cultured in DMEM high glucose (GE Healthcare Life Sciences) (4.5g/l) supplemented with 10% heat-inactivated foetal bovine serum (LabTech International), penicillin (100 units/ml) and streptomycin (100 units/ml), 2 mM glutamine (Invitrogen) and non-essential amino acids (Sigma). Cells were transfected with plasmids using Fugene-6 (Promega) and siRNAs using Oligofectamine (ThermoFisher Scientific) according to the manufacturer's instructions. Briefly, cells were plated in 12 well plates and transfected 24 h later with 1 μg of the plasmid of interest or 100 nM siRNAs. For double transfections, 0.5 μg of each plasmid was used. Transfected cells were analysed 24 h and siRNA treated cells 72 h post transfection. For experiments involving Mito-RFP-ER, cells were transfected with 0.2 μg of each plasmid and analysed 16 h post transfection. For induction of autophagy, cells were treated with 100 nM Rapamycin for 12 hours or 500 nM Torin-1 for 1 hour. Starvation induced autophagy involved culture in Earle's balanced salt solution (EBSS) (Sigma) for 2h. For autophagy flux assays, cells were treated with 400 nM Bafilomycin A1 for 4 hours. Cells were treated with 2 μM Xestospongin C for 12 h and 5 μM Ruthenium-360 for 2 h. EGFP-LC3 stably transfected HeLa were generated by selection with 1 mg/ml G418 (Sigma) for 28 days.

SDS-PAGE and immunoblotting

Cells were harvested by washing in ice-cold phosphate buffered saline (PBS) and then scraped into PBS containing 1% SDS with protease inhibitors (Complete Roche), 1 mM Na_3VO_4 and 5 mM NaF. Extracts were then heated for 5 min at 100°C, sonicated and centrifuged at 10000 g (av) for 10 min. Protein concentrations were determined using a commercial BCA assay (Pierce). Samples were prepared for SDS-PAGE by addition of sample buffer and then resolved on 10 or 15 % SDS-PAGE gels, and transferred to nitrocellulose membranes (Schleicher & Schuell Bioscence) by wet electroblotting (BioRad). Membranes were blocked with Tris-HCl-buffered saline (TBS) containing 5% dried milk and 0.1% Tween-20 for 1 h at 20°C, and then incubated with primary antibodies in TBS containing 5% bovine serum albumin/0.1% Tween-20 for 16 hours at 4°C. Following washing in TBS containing 0.1% Tween-20, the blots were incubated with horseradish peroxidase conjugated secondary antibodies and developed using chemiluminescence with a Luminata Forte Western HRP substrate system according to the manufacturer's instructions (Millipore). Detection of chemiluminescence signals was by using a BioRad ChemiDoc MP Imaging system or X-ray films.

Signals on films were quantified using ImageJ after scanning with an Epson Precision V700 Photo scanner essentially as described by us in previous studies [S4].

Immunofluorescence microscopy

Cells grown on coverslips were fixed for 15 min at 20°C with 4% (w/v) paraformaldehyde in PBS and then permeabilized with PBS containing 0.5% Triton X-100 for 15 minutes. Samples were then preincubated with blocking buffer (PBS containing 2% horse serum and 0.5% Triton X-100) for 1 hour and incubated with primary antibodies diluted in blocking buffer for 1 hour. Following washing in PBS containing 0.5% Triton X-100, the samples were incubated with goat anti-rabbit or goat anti-mouse AlexaFluor-488 or -594-conjugated Igs in PBS for 1 hour, washed in PBS and then mounted in Vectashield mounting medium containing DAPI (Vector Laboratories).

Proximity ligation assays to quantify IP3 receptor3-VDAC1 interactions were performed essentially as described previously [S1] using Duolink reagents (Sigma-Aldrich). Cells were fixed in 4% paraformaldehyde in PBS and probed with rabbit IP3 receptor3 and goat anti-VDAC1 antibodies. Signals were developed using a Duolink In Situ Far Red kit and quantified using NIH ImageJ.

Images were acquired using a Leica TCS-SP5 confocal microscope using a ×63HCX PL APO lambda blue CS 1.4 oil UV objective. Images were collected using single excitation for each wavelength separately (488 nm Argon Laser line and a 500–545 nm emission band pass; 561 nm DPSS Laser line and a 585–690 nm emission band pass; 405 nm UV diode and a 422–470 nm emission band pass). Ten to fifteen image sections of selected areas were acquired using a pinhole size of one Airy unit. Z-stack images were analysed and processed using Leica Applied Systems (LAS AF6000) image acquisition software. The same laser intensity was used for image acquisition in each experiment. Conventional wide-field immunofluorescence microscopy was performed with a Leica DM5000B microscope equipped with 63×/1.25NA HCX-PL-FLUOTAR objectives and appropriate filtersets (Leica Microsystems).

Quantitative image analysis

EGFP-LC3 and EGFP-DFCP1 structures in cells were quantified essentially as previously described [S5]. For HeLa cells a threshold of 20 dots/cell was established using control treatments that effectively distinguishes "autophagy active" and "autophagy inactive" as described [S6]. Hence, the percentage of cells displaying more than 20 punctae were quantified; see [S6]. EGFP-HDQ74 aggregation was quantified by determining the proportion of EGFP-HDQ74 transfected cells that contained aggregates again as previously described [S7]. In this assay, cells were excluded from quantification if the DAPI-stained nuclei showed apoptotic morphology (fragmentation or pyknosis). Proximity ligation assays signals, ULK1 and ATG5 structures were analysed using NIH ImageJ macro "EGFP-LC3" [S8].

Ca^{2+} measurements

Ca^{2+} measurements were performed essentially as described previously [S1, S2, S9]. HEK293 cells were transfected with M3R plus experimental plasmids for 24 h and then loaded with 2 μM Rhod2-AM or Fluo4-AM dye (Invitrogen) in external solution (145 mM NaCl, 2 mM KCl, 5 mM $NaHCO_3$, 1 mM $MgCl_2$, 2.5 mM $CaCl_2$, 10 mM glucose, 10 mM Na-HEPES, pH 7.25) containing 0.02% Pluronic-F27 (Invitrogen) for 15 min at 37°C. Rhod2 and Fluo4 fluorescence were timelapse recorded (1s intervals) at 37°C with MetaMorph (Molecular Dynamics) on an Axiovert S100 microscope (Zeiss) equipped with DsRed (Rhod2) and GFP (Fluo4) filtersets (Chroma Technology), a 40x Plan-Neofluar 1.3NA objective (Zeiss), and a Photometrics Cascade-II 512B36 EMCCD camera. Some experiments were also performed using a Nikon Eclipse TiE microscope with a CFI Plan Fluor 40x oil N.A. 1.30 W.D. 0.2 mm spring loaded lens, TiND6 PFS-S Perfect Focus Unit, Chroma filtersets and Bio-Logic MSC-200 fast perfusion system; images were acquired using an Andor Neo sCMOS camera and data analysed using Nikon proprietary software. The cells were kept under constant perfusion with external solution (0.5 ml min^{-1}). IP3 receptor-mediated Ca^{2+} release from ER stores was triggered by application of 100 μM oxotremorine-M for 2 min. Mitochondrial calcium levels were then calculated as relative Rhod2 or Fluo4 fluorescence compared to baseline fluorescence at the start of the measurement.

Electron microscopy

HeLa cells were fixed with 2% glutaraldehyde in 0.1 M sodium cacodylate buffer (pH 7.2) for 30 minutes and then harvested by scraping gently with a plastic scraper. The cells were pelleted by centrifugation at 800g (av) for 10 min and fixed for a further 2 hours in 2% glutaraldehyde in 0.1 M sodium cacodylate buffer. Following washing in buffer, the cells were post-fixed for 1 hour in 1%

osmium tetroxide in 0.1 M sodium cacodylate buffer. The cells were then stained for 1 hour with 1% uranyl acetate in water before dehydration and embedding in Taab resin. Sections were cut using a Reichert Ultra cut E ultramicrotome and stained for 6 minutes in 0.16% lead citrate in 0.1 M NaOH followed by 3 washes in water. Samples were viewed on a Tecnai 20 electron microscope at 4800X and 1900X magnification. Digital images were acquired and autophagic structures identified via morphology as described [S10]. Quantification of autophagic structures per cell area was performed using Image J (plug-in Grids and Cell Counter; NIH, Bethesda, MD, USA) using point counting as described [S11].

Statistical analyses

All experiments were repeated at least three times. Statistical analyses were performed with Prism 5.0 (GraphPad Software). For quantification of LC3-II levels, signals were normalized to α-tubulin as a housekeeping loading control as recommended [S12].

References

S1. De Vos, K.J., Morotz, G.M., Stoica, R., Tudor, E.L., Lau, K.F., Ackerley, S., Warley, A., Shaw, C.E., and Miller, C.C.J. (2012). VAPB interacts with the mitochondrial protein PTPIP51 to regulate calcium homeostasis. Hum. Mol. Genet. *21*, 1299-1311.

S2. Stoica, R., De Vos, K.J., Paillusson, S., Mueller, S., Sancho, R.M., Lau, K.F., Vizcay-Barrena, G., Lin, W.L., Xu, Y.F., Lewis, J., et al. (2014). ER-mitochondria associations are regulated by the VAPB-PTPIP51 interaction and are disrupted by ALS/FTD-associated TDP-43. Nat. Commun. *5*, 3996.

S3. Csordas, G., Renken, C., Varnai, P., Walter, L., Weaver, D., Buttle, K.F., Balla, T., Mannella, C.A., and Hajnoczky, G. (2006). Structural and functional features and significance of the physical linkage between ER and mitochondria. J. Cell Biol. *174*, 915-921.

S4. Morotz, G.M., De Vos, K.J., Vagnoni, A., Ackerley, S., Shaw, C.E., and Miller, C.C.J. (2012). Amyotrophic lateral sclerosis-associated mutant VAPBP56S perturbs calcium homeostasis to disrupt axonal transport of mitochondria. Hum. Mol. Genet. *21*, 1979-1988.

S5. Gomez-Suaga, P., Luzon-Toro, B., Churamani, D., Zhang, L., Bloor-Young, D., Patel, S., Woodman, P.G., Churchill, G.C., and Hilfiker, S. (2012). Leucine-rich repeat kinase 2 regulates autophagy through a calcium-dependent pathway involving NAADP. Hum. Mol. Genet. *21*, 511-525.

S6. Mizushima, N., Yoshimori, T., and Levine, B. (2010). Methods in mammalian autophagy research. Cell *140*, 313-326.

S7. Ravikumar, B., Imarisio, S., Sarkar, S., O'Kane, C.J., and Rubinsztein, D.C. (2008). Rab5 modulates aggregation and toxicity of mutant huntingtin through macroautophagy in cell and fly models of Huntington disease. J. Cell Sci. *121*, 1649-1660.

S8. Dagda, R.K., Zhu, J., Kulich, S.M., and Chu, C.T. (2008). Mitochondrially localized ERK2 regulates mitophagy and autophagic cell stress: implications for Parkinson's disease. Autophagy *4*, 770-782.

S9. Stoica, R., Paillusson, S., Gomez-Suaga, P., Mitchell, J.C., Lau, D.H., Gray, E.H., Sancho, R.M., Vizcay-Barrena, G., De Vos, K.J., Shaw, C.E., et al. (2016). ALS/FTD-associated FUS activates GSK-3beta to disrupt the VAPB-PTPIP51 interaction and ER-mitochondria associations. EMBO Rep. *17*, 1326-1342.

S10. Eskelinen, E.L. (2008). To be or not to be? Examples of incorrect identification of autophagic compartments in conventional transmission electron microscopy of mammalian cells. Autophagy *4*, 257-260.

S11. Yla-Anttila, P., Vihinen, H., Jokitalo, E., and Eskelinen, E.L. (2009). Monitoring autophagy by electron microscopy in Mammalian cells. Methods Enzymol. *452*, 143-164.

S12. Klionsky, D.J., Abdalla, F.C., Abeliovich, H., Abraham, R.T., Acevedo-Arozena, A., Adeli, K., Agholme, L., Agnello, M., Agostinis, P., Aguirre-Ghiso, J.A., et al. (2012). Guidelines for the use and interpretation of assays for monitoring autophagy. Autophagy *8*, 445-544.
